# Determining Exposure Factors of Anti-Fogging, Dye, Disinfectant, Repellent, and Preservative Products in Korea

**DOI:** 10.3390/ijerph15020232

**Published:** 2018-01-30

**Authors:** Daeyeop Lee, Joo-Hyon Kim, Taksoo Kim, Hyojung Yoon, Areum Jo, Byeongwoo Lee, Hyunwoo Lim, Pilje Kim, Jungkwan Seo

**Affiliations:** Division of Risk Assessment, National Institute of Environmental Research, Hwangyeong-ro 42, Seo-gu, Incheon 22689, Korea; daeyub22@snu.ac.kr (D.L.); jhkim0318@korea.kr (J.-H.K.); taksoo@korea.kr (T.K.); hyojay97@korea.kr (H.Y.); jojo721@korea.kr (A.J.); dreaming0628@korea.kr (B.L.); yaho365@korea.kr (H.L.); newchem@korea.kr (P.K.)

**Keywords:** consumer products, exposure factor, exposure assessment, risk-concerned products

## Abstract

Reliable exposure factors are essential to determine health risks posed by chemicals in consumer products. We analyzed five risk-concerned product categories (anti-fogging, dye, disinfectant, repellent, and preservative products) for 13 products (three car anti-fogging products, a lens anti-fogging product, two car dye products, two drain disinfectants, an air conditioner disinfectant, a chlorine-based disinfectant, a fabric repellent, an insect repellent for food, and a wood preservative) considered to be of high risk in order to determine exposure factors via web surveys and estimation of amount of product. Among the 3000 participants (1482 (49%) men) aged ≥19 years, drain disinfectants were used most frequently (38.2%); the rate of usage of the other products ranged between 1.1–24.0%. The usage rates for the consumer products differed by sex, age, income, and education. Some consumer products such as car and lens anti-fogging products, chlorine-based disinfectants, fabric repellents, and drain disinfectants were regularly used more than once a month, while car dye products, air conditioner disinfectants, insect repellents for food, and wood preservatives were not regularly used owing to the specific product purposes and seasonal needs. Our results could be used for managing or controlling chemical substances in consumer products and conducting accurate exposure assessments.

## 1. Introduction

People commonly use a variety of consumer products for household cleaning and personal care, as they improve sanitary conditions in daily life. However, consumer products are composed of various chemical substances, which act as active ingredients, solvents, preservatives, and additives. There is a growing concern that people are inevitably exposed to multiple chemicals through the use of consumer products [1,2]. Previous studies have reported that chemicals in consumer products may induce adverse health effects; some of these chemicals are known to cause skin rashes, allergies, eye irritation, and respiratory irritation [3]. Additionally, internal exposure to chemicals that can be found in consumer products has been associated with cancer, endocrine disruption, birth defects, and allergic dermatitis [4,5,6,7].

In Korea, the potential adverse effects of consumer products were highlighted when a consumer applied disinfectants to a humidifier to sterilize a water tank. In 2011, an unidentified case of fatal lung disease was reported, probably caused by the hazardous chemicals present in these disinfectant products [8]. According to the Korean Ministry of Environment (MOE), as of 13 January 2017, approximately 200 deaths and 700 injuries have reported as a result of this type of potential exposure [9]. After the specified incident, public concern regarding the use of chemicals in products increased. Therefore, in order to mitigate people’s concerns regarding the use of chemicals in consumer products, exposure and health risks posed by the use of consumer products should be assessed.

To conduct exposure assessments of consumer products, information on exposure factors (e.g., frequency of use, amount used per application, and information about the circumstances of usage) are necessary [10]. Several studies on exposure factors associated with consumer products were conducted in Europe and the USA. In Europe, the Exposure Patterns and Health Effects of Consumer Products (EPHECT) project was set up as a European consumer product database [11]. The EPHECT conducted exposure and risk assessments for chemicals contained in consumer products [12,13]. In the USA, the Study of Use of Products and Exposure-Related Behaviors (SUPERB) project provided data on usage patterns for many consumer products [14].

Exposure factors may vary by country [15,16,17]. A national database of exposure factors for consumer products is essential to conduct exposure and risk assessments. The National Institute of Environmental Health (NIER) conducted a study to collate an exposure factor database for consumer products [18]. Moreover, a previous study conducted in Korea determined the exposure factors for 10 consumer products (face cleaner, toothpaste, shampoo, hair conditioner, body wash, dish and laundry detergents, fabric deodorizer, antistatic spray, and shoe polish) [17]. In April 2015, the MOE enforced regulations that designated the following eight product categories as risk-concerned products, which are defined as being any chemical product declared by the MOE as one that is likely to cause risk to humans or the environment. These include cleaning products, adhesives, coating products, bleaching products, air fresheners, deodorizers, detergents, and fabric softeners [19]. These regulations were based on the exposure factors determined for all eight product categories [17,18]. However, by June 2015, the number of risk-concerned products increased, with the addition of the following seven product categories: anti-rust additives, anti-fogging products, dye products, disinfectants, repellents, preservatives, and tattoo inks [20]. Only a few studies have investigated the exposure factors for these product categories, and hence, further studies are needed.

The purpose of this study was to develop a national representative database of exposure factors for the risk-concerned products newly identified in June 2015 in Korea. To obtain information on exposure factors, 13 products were investigated for frequency of use, amount used per application, and daily consumption of products. These exposure factor data determined in this study could be useful in establishing safety guidelines for products and conducting accurate exposure and risk assessments.

## 2. Methods

### 2.1. Study Population

A total of 3000 people were selected for inclusion in this study in order to obtain a substantial number of users for each product. To ensure that the study population was representative of the actual product users in Korea, the exposure factor survey was conducted in 15 metropolitan areas and provinces, including rural areas in Korea. The number of participants included in each province was based on the proportional population of the province relative to the total population, while considering sex and age balances. Within each province, survey locations were randomly selected, and for each survey location, the number of participants was fixed for each metropolitan area. When a participant did not want to participate in this study, new participants were recruited until the required number of participants in each survey location was reached.

### 2.2. Data Collection

#### 2.2.1. Web Survey

A web questionnaire survey was administered between June and July 2015. Before starting the web survey, an e-mail was sent to potential participants. The email addresses were obtained from a national survey company and an online shopping voucher was offered in support of participation in the study. If the participant agreed to take the survey, a web link was sent to them in order to start the survey. All subjects gave their informed consent for inclusion before they participated in the study. This web survey collected current information on the exposure factors for five product categories (anti-fogging, dye, disinfectant, repellent, and preservative products) and 13 products (three car anti-fogging products (liquid, tissue, and film), a lens anti-fogging product (tablets), a fabric repellent (trigger spray), an insect repellent for food (tablets), and a wood preservative (aerosol spray)).

**Figure 1:**
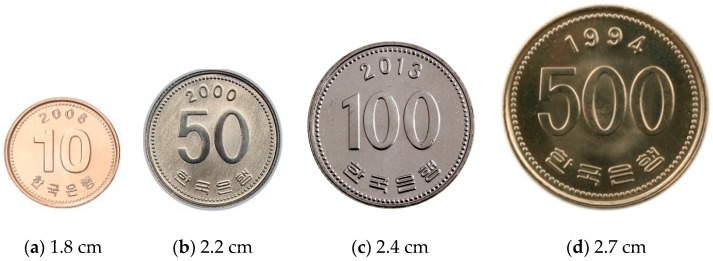
Examples of four coins of different sizes (diameter 1.8–2.7 cm) displayed in the questionnaire to assess the amounts of applied product (pen type).

The questionnaire collected demographic information (e.g., address, age, sex, income per month, and education level), frequency of use, amount used per application, duration of use, and protective action (e.g., ventilation, use of gloves, a mask, and/or the instruction manual). Participants had to select from four options (e.g., weekly, monthly, six-monthly, and yearly), and then fill the frequency of use (e.g., 2 times/week) by selected duration. Participants additionally had to indicate the amount used per application. Different questions were used for the specific type of application. For trigger sprays, the number of squeezes per application was determined. For aerosol sprays, the spraying time per application was queried. For liquid types, the number of products used was estimated with reference to a 50-mL glass, the most popular glass size used for liquid in Korea [17]. For pen types, four differently sized Korean coins (diameter 1.8–2.7 cm) were displayed with photographs (Figure 1), and then, participants were required to select one of them. For tissue, film, and tablet types, the number of each product used per application was determined.

For validation of the questionnaire, if a participant chose extreme values or left several questions blank, an additional questionnaire was conducted. After that, the questionnaire was validated (per the difference between the first and second questionnaire). If there was a large difference, the participants were excluded and new participants were recruited.

#### 2.2.2. Product Amount Estimation

Experiments were conducted to estimate the accurate amount of product used per application. The 13 products included among the target products in this study were purchased online and from supermarkets based on the sales rankings at a large Korean shopping mall (e.g., e-mart mall and Lotte shopping mall). The products purchased were assumed to be commonly used by Korean people. Mass generation (g/s) or amounts of products used (g/unit or g/new product) were estimated by application type. To estimate mass generation for spray products (trigger and aerosol spray), a report from the Dutch National Institute for Public Health and the Environment (RIVM) was used as a reference [21]. In the report, the mass generation rate was estimated by squeezing the product trigger 10 times for about 6 s (trigger spray) or spraying the product for 10 s (aerosol spray); the product weight was measured before and after product use [21]. In this study, for trigger spray, mass generation was estimated by squeezing the product 10 times and measuring spray time, while for aerosol spray, the method used in the RIVM report was employed.

For liquid products, the volume of the product used was measured using a 50-mL glass, the most popular glass size used for liquid in Korea. For pen products, the differences in the mass before and after use of the product were measured by using depictions of four coins of different sizes (Figure 1). To check for data accuracy, the product amount estimation for individual products were conducted in triplicate. For tablet products, the concentration of new products was measured, and for tissue and film products, the amount could not be estimated because of very small differences before and after use of the products.

#### 2.2.3. Definitions

For the purpose of this study, participants were stratified into three age groups: young (19–29 years), middle-aged (30–49 years), and older (≥50 years). Additionally, monthly income was categorized into three groups: low (<$USD 2000), middle ($USD 2000–4000), and high (>$USD 4000). Finally, participant education levels were categorized as follows: low (middle school or less), medium (high school), and high (college or greater). The product use rate was defined as the proportion of participants who reported the use of consumer products at least once in the last 12 months.

### 2.3. Data Analysis

All statistical analyses were conducted using R version 3.3.2 (R Foundation for Statistical Computing, Vienna, Austria). Frequency tables were constructed to assess the rate of product use. Chi-square tests were used to analyze the differences in the rates of usage based on sex, age, education, and income. The frequency and duration of use were calculated using data from the web survey. The amount used per application (g/use) was calculated by multiplying the use patterns (e.g., spraying time, number or product use, and amount of product) obtained from the web survey by mass generation or amount of product obtained from the estimation of procedure. The consumption per day (g/day) was calculated by multiplying the individual frequency of use (use/month) by the individual amount of use per application (g/use) and the conversion factor (month/30 days). *p* < 0.05 was considered to indicate statistical significance.

## 3. Results

### 3.1. Study Population Demographics

In total, 3000 participants completed the survey, of whom 50.6% (*n* = 1518) were women. Overall, 25.2%, 63.4%, and 11.4% of the participants were young, middle-aged, and older respectively. Additionally, 12.5%, 35.0%, and 52.5% of the participants were classified as belonging to low-, middle-, and high-income levels, respectively (Table 1).

Finally, 1.0%, 18.4%, and 80.6% of the participants had low, medium, and high educational levels, respectively. The proportion of individuals in each income group did not significantly differ based on sex (*p* = 0.25), whereas the proportions of individuals in each educational and age group significantly differed by sex (*p* < 0.01). Among men, the middle-aged group was the largest (67.6%) and the younger and older groups comprised 17.9% and 14.5% of participants, respectively. Among women, the younger and older groups comprised 32.4% and 8.3% of participants, respectively, and the middle-aged group was the largest (59.3%; Table 1).

### 3.2. Product Use Rate

Among the anti-fogging products, car anti-fogging products were used more frequently than lens anti-fogging products. Car anti-fogging products, were more commonly used by men than by women (*p* < 0.01). Participants in the elderly group used anti-fogging products most frequently (*p* < 0.05). Moreover, the higher earning and more educated participants were more likely to use anti-fogging products than those categorized as belonging to low and middle/medium income or education levels (*p* < 0.05; Table 2).

Dye products were used by 14.9% of the participants. Men used car colorant products more often than women (*p* < 0.01). Like anti-fogging products, the older age group used car dye products most frequently. Although higher earning participants were more likely to use car dye products than the low and medium income groups, those in the low education group used these products most frequently (*p* < 0.05).

The rate of disinfectant product use ranged 5.6–38.2%. With the exception of chlorine-based disinfectants, the use of disinfectants significantly differed by sex. Women were more likely to use drain disinfectants (*p* < 0.01). However, men were more likely to use air conditioner disinfectants (*p* < 0.01). The rate of air conditioner and chlorine-based disinfectant use was higher among the older group than the other age groups (*p* < 0.05). For all disinfectant products, participants who earned more were more likely to use disinfectant products (*p* < 0.01). Furthermore, for drain disinfectant product and air conditioner product, highly educated participant more likely to use the products (*p* < 0.05).

The use of repellent products ranged 7.9–9.7% and did not significantly differ by sex. The older and higher earning participants were more likely to use fabric repellent products; there were no significant differences based on educational levels. There were no significant differences in the use of insect repellent for food by age, income, or education. Only 1.1% of participants used preservatives, and there were no significant differences observed based on sex, age, and income and educational levels in terms of the use of preservatives.

### 3.3. Frequency of Use

**Figure 2:**
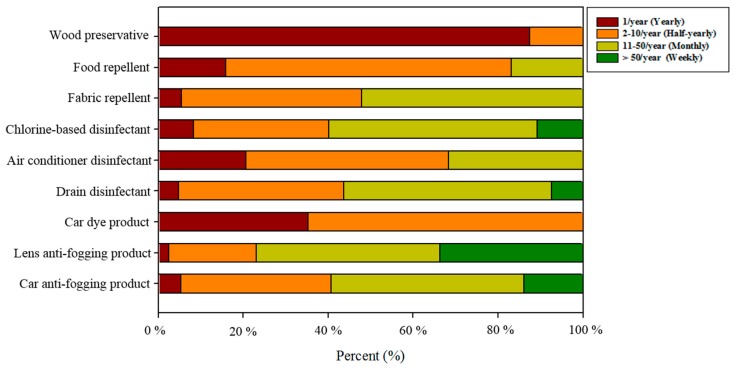
Frequency of use by product.

Figure 2 presents the frequency of use of each product. None of the products investigated in this study were for daily use, and the frequency of use varied by the product. The frequency of use of anti-fogging products differed according to the purpose of use. In total, 76.9% and 59.2% of participants used lens and car anti-fogging products more than once a month (weekly and monthly), with mean frequencies of 5.6 and 2.4 times per month, respectively. Only a few participants used car dye products more than once a month: the mean frequency of use was 0.2 times per month (Figure 2). Among disinfectant products, drain and chlorine-based disinfectants were used more frequently than air conditioning disinfectants. In total, 56.3% and 59.9% of participants used drain disinfectants and chlorine-based disinfectants more than once a month, respectively; the mean frequencies of use were 1.8 and 2.7 times per month, respectively. Air conditioning disinfectants were typically used before and after the air conditioner operating seasons; the mean frequency of use was 0.2 times per month. Among repellent products, all types of products were used less than 50 times per year, with means ranging 0.4–1.0 times per month. Wood preservative products were used less than once a month, with a mean frequency of use of 0.1 time per month. 

### 3.4. Usage Pattern of Consumer Products

Lens anti-fogging products (tissue) were used most frequently among the participants of this study, whereas wood preservatives (aerosol) were rarely used (Table 3). In general, the amount used per application differed for the same product. For disinfectant products, chlorine-based disinfectants (tablet) were used in the greatest quantity per application, followed by air conditioner (aerosol) and drain (tablet) disinfectants, while drain disinfectants (trigger) were used in the lowest quantities. However, the rank order of amount used per day differed. Drain (trigger) and air conditioner (aerosol) disinfectants demonstrated the lowest quantities used on a daily basis.

Participants were asked how long it took them to use the product from beginning to end. In general, the duration of use for aerosol and trigger sprays was longer than that for tissue and tablet products. Among all products, car anti-fogging products (film) had the longest duration of use, while fabric and tablet repellents (both tablets) had the shortest duration of use.

### 3.5. Protective Actions with Products

Participants were asked if they wore gloves or masks, ventilated rooms, and/or read product instruction manuals as protective measures. Seventy five percent of the participants used gloves when they used wood preservative products (aerosol), whereas for repellent products, the proportions of participants who used gloves ranged 13.4–22.5%. In general, when the participants used trigger or aerosol sprays, they were more likely to use masks and ventilate the room. Over 50% of participants claimed to read product instruction manuals for the majority of the products (Table 4).

## 4. Discussion

The aim of this study was to create a national exposure factor database for risk-concern products designated by the MOE in June 2015. This study focused on the frequency of use and amount of use at each application, which is necessary for accurate exposure assessments.

In April 2015, the MOE in Korea enforced its Notice No. 2015-41, setting regulations for safety and labelling standards for eight product categories designated as risk-concerned products. These products included the following: cleaning products, detergents, bleach, fabric softeners, coating products, air fresheners, adhesives, and deodorants [19]. At that time, several consumer products were managed by the Ministry of Trade, Industry, and Energy in Korea (MOTIE). However, in June 2015, the MOE revised the regulations for the safety and labeling standards for risk-concerned products. Seven product categories (anti-rust additives, anti-fogging and dye products, disinfectants, repellents, preservatives, and tattoo inks), which were previously managed by MOTIE, were transferred to the MOE [20]. In order to assess the health risks, the MOE needed information on the use of these newly added risk-concerned products. Therefore, anti-fogging and dye products, disinfectants, repellents, and preservatives were selected as target consumer products among the newly added risk-concerned products identified in this study.

In previous studies that investigated exposure factors for household and personal care products, the actual users of the consumer products were considered when recruiting participants [11,15,22]. In this study, all the target consumer products were commonly used by adults, and therefore, all individuals included in this study were aged 19 years and older.

To obtain sufficient numbers of participants who consumed each product, 3,000 participants were recruited for inclusion in this study. One study found that as sample sizes increased, the exposure factors became more similar to those of the parent population for the majority of the consumer products, and a sample size of >50–100 was recommended for each product [23]. Based on these recommendations, at least 50 participants were enrolled to accurately represent all the consumer products. However, only 32 participants used preservative products. Therefore, the error in estimating the exposure factors for preservative factors should be considered in any further calculations.

The frequencies of usage for the products investigated differed by country (Table 5). Insect repellents, including insect repellent for food, were used much more frequently in California than in Korea; only 9.7% of the participants used insect repellents for food in this study, while approximately 50% used them in California [14].

This study reported that trigger and tablet disinfectants in Korea were used more than twice a month (Table 5). However, another study reported that drain disinfectants are used less than once a month [24]. The participants of this study used insect repellent for food less than once a month. However, in California, people used it approximately twice a month [14], and the RIVM reported that people used it more than four times a month [25]. Wood preservative products were found to be used approximately once a year.

In general, when consumers use spray or liquid products, they might use gloves, a mask, or ventilate the area due to dermal contact and odor. A study investigated protective action taken to reduce exposure during use of cleaning, automotive care, and surface protection products [22]. In that study, except for household bleach (spray and liquid), the usage rates of gloves, masks, and ventilation were under 70%, 6.5%, and 45.0%, respectively. In this study, for chlorine-based disinfectants (tablet), the usage rates of gloves, masks, and ventilation were 68.3%, 28.1%, and 79.0%, respectively. As this was an online survey, the participants’ comprehension of the questions posed might have impacted the quality of the data; in essence, in-person surveys might yield better quality data than web surveys [26,27].

Several studies have examined personal hygiene and cosmetic product exposure factors [14,15,28,29], and exposure factors have been compared in various studies [15,16,17]. However, only a few studies have focused on home care products. Therefore, it was difficult to compare exposure factors between countries.

To protect people from hazardous chemicals in consumer products, exposure and risk assessment are crucial tools. In particular, exposure assessment is a vital step of risk assessment for people exposed to hazardous chemicals from using consumer products. To estimate chemical exposure, the deterministic method is widely used [12,30,31]. When many researchers used the deterministic method, exposure factors (e.g., amount of use, frequency, and duration of use) were used as input parameters. The deterministic method may over- and underestimate exposure level due to extreme values [32]. In Korea, before the national exposure factors were disseminated, the input parameters from the RIVM were widely used. As several studies have already reported, exposure factors differ by country, race, and other factors [15,16,22]. Therefore, a national exposure database is needed to understand a country’s own consumer product exposure. Based on the results of this study, exposure factors for 13 products were established in the regulation methods for risk assessment for risk-concerned products [33]. There were several strong points. First, the exposure factors were set up for the 5th, 50th, 75th and 95th percentile values. Hence, researchers could choose the values by product characteristics and consumer use (e.g., heavy user) and conduct realistic exposure assessments. Second, the exposure factors were uploaded on the NIER website. Thus, when the government, researchers, and manufacturers conduct exposure and risk assessments, they can use these data to conduct accurate exposure and risk assessments. Third, the government (e.g., MOE) could set up safe guidelines in line with the data regarding the Korean consumer usage pattern. For example, to prevent risks from exposure to consumer products, reasonable concentrations of the risk chemicals in these products could be established according to realistic exposure assessments.

There were some limitations. In this study, potential seasonal variations in product use were not considered; therefore, the frequency of use or amount used might have been affected by the study period. However, disinfectant products, repellent products, and preservative products are used more frequently in summer than in winter. Conducting surveys in summer guarantees that the exposure factors of these products were not underestimated. Face-to-face surveys often generate data of superior quality compared to that with web surveys. However, owing to the magnitude of the participant numbers enrolled, as well as a limited budget, it was not feasible to conduct in-person surveys. We believe that since the participants were aged ≥19 years, the frequency of use and amount of the products use per application could be recorded.

## 5. Conclusions

This study investigated recent exposure factors for five categories and 13 products commonly used in daily life in Korea. The exposure factors were determined via a web survey including over 3000 people in Korea. The result showed that exposure factors can be different for the same product based on application. Therefore, when exposure assessments are conducted, type of application must be considered. The exposure factors investigated in this study can be used for managing or controlling chemical substances in consumer products and conducting accurate exposure assessments. The number of consumer products used during one’s lifetime have increased; therefore, further research on exposure factors for consumer products should be conducted.

## Figures and Tables

**Table 1:** Demographic characteristics of the study population.

Variable	Level	*n*	%
Sex	Men	1482	49.4
	Women	1518	50.6
Age	Young (15–34 years)	757	25.2
	Middle-aged (35–49 years)	1902	63.4
	Older (≥50 years)	341	11.4
Income	Low (<$USD 2000)	375	12.5
	Middle ($USD 2000–4000)	1051	35.0
	High (>$USD 4000)	1574	52.5
Education	Low (middle school or less)	31	1.0
	Medium (high school)	552	18.4
	High (college or greater)	2417	80.6

**Table 2:** Use of risk-concerned products (%) stratified by sex, age, income, and education levels.

Category	Product	Overall	Sex		Age			Income			Education		
			Men	Women	Young	Middle-Aged	Older	Low	Middle	High	Low	Medium	High
		*n*	1482	1518	757	1902	341	375	1051	1574	31	552	2417
Anti-fogging	Car anti-fogging	24.0%	29.8% **	18.4% **	13.5% **	26.1% **	35.8% **	11.2% **	19.6% **	30.0% **	9.7% **	19.7% **	25.2% **
	Lens anti-fogging	5.3%	6.1%	4.6%	4.4% **	5.2% **	8.2% **	1.9% **	3.8% **	7.2% **	3.2% *	3.3% *	5.8% *
Dye	Car colorant	14.9%	20.6% **	9.4% **	9.4% **	16.1% **	20.8% **	9.6% **	11.1% **	18.7% **	16.1% *	11.1% *	15.8% *
Disinfectant	Drain disinfectant	38.2%	34.8% **	41.5% **	35.9%	39.3%	37.0%	28.3% **	36.1% **	41.9% **	25.8% *	34.4% *	39.2% *
	Air conditioner disinfectant	19.5%	21.9% **	17.1% **	14.1% **	20.3% **	26.1% **	12.5% **	15.6% **	23.8% **	9.7% *	15.9% *	20.4% *
	Chlorine-based disinfectant	5.6%	5.7%	5.4%	5.0% *	5.2% *	8.8% *	3.7% **	4.1% **	7.0% **	3.2%	4.7%	5.8%
Repellent	Fabric repellent	7.9%	7.8%	8.0%	5.4% *	8.5% *	9.7% *	4.0% **	5.9% **	10.1% **	6.5%	7.2%	8.0%
	Insect repellent for food	9.7%	8.8%	10.5%	8.3%	10.0%	10.9%	6.7% **	8.4% **	11.2% **	9.7%	8.7%	9.9%
Preservative	Wood preservative	1.1%	1.1%	1.0%	1.3%	0.9%	1.5%	1.3%	0.8%	1.2%	3.2%	1.4%	1.0%

* *p* < 0.05, ** *p* < 0.01.

**Table 3:** Summarized statistics (median and interquartile range *) of exposure factors by product and application type.

Category	Product	Application Type	Frequency of Use (use/month)	Amount of Use per Application (g/use)	Amount of Use per Day ** (g/day)	Duration of Use (min)
Anti-fogging	Car anti-fogging	Liquid	1.0 (0.3–12.0)	2.7 (2.0–5.5)	0.1 (0.02–2.2)	5.0 (1.0–23.6)
		Tissue	1.0 (0.2–8.0)	2.0 (1.0–5.0) *	N/A	5.0 (1.0–15.0)
		Film	0.2 (0.08–0.2)	1.0 (1.0–2.0) *	N/A	30.0 (30.0–55.0)
	Lens anti-fogging	Tissue	4.0 (0.2–20.2)	1.0 (1.0–3.1) *	N/A	1.5 (0.3–5.0)
Dye	Car dye	Aerosol	0.2 (0.1–0.5)	7.4 (1.7–27.2)	0.05 (0.01–0.5)	10.0 (1.0–50.0)
		Pen	0.2 (0.1–0.5)	0.003 (0.0003–0.02)	0.00002 (0.000001–0.003)	5.0 (1.0–30.0)
Disinfectant	Drain disinfectant	Trigger	1.0 (0.2–8.0)	5.0 (1.2–15.9)	0.2 (0.01–4.2)	5.5 (1.0–31.6)
		Tablet	0.8 (0.1–3.0)	17.0 (15.7–34.1)	0.5 (0.05–3.4)	1.3 (0.2–5.0)
	Air conditioner disinfectant	Aerosol	0.3 (0.1–2.0)	22.3 (4.5–89.0)	0.2 (0.02–5.9)	10.0 (1.0–30.0)
	Chlorine-based disinfectant	Tablet	1.0 (0.1–12.0)	100.6 (50.3–402.4)	3.4 (0.2–161.0)	20.0 (10.0–30.0)
Repellent	Fabric repellent	Trigger	1.0 (0.1–2.0)	16.0 (0.7–19.0)	0.5 (0.002–1.3)	3.0 (1.0–10.2)
	Insecticide for food	Tablet	0.2 (0.1–1.0)	6.1 (6.1–12.3)	0.04 (0.02–0.4)	1.0 (0.5–5.0)
Preservative	Wood preservative	Aerosol	0.1 (0.1–0.2)	6.7 (2.3–11.7)	0.02 (0.01–0.1)	20.3 (5.0–30.2)

* The number of product use (wipe or film type). ** Amount of use per day (g/day) = Frequency of use (use/month) × Amount of use per application (g/use) × Conversion factor (month/30 days).

**Table 4:** Protective actions taken to reduce exposure during product use.

Category	Product	Application Type	Glove	Mask	Ventilation	Instruction Manual
Anti-fogging	Car anti-fogging	Liquid/Tissue/Film	33.3%	11.1%	56.4%	53.0%
	Lens anti-fogging	Tissue	27.5%	12.5%	-	51.3%
Dye	Car colorant	Aerosol	56.3%	27.3%	-	58.8%
		Pen	34.2%	6.8%	-	46.9%
Disinfectant	Drain disinfectant	Trigger	49.2%	14.5%	73.6%	57.5%
		Tablet	26.0%	5.2%	-	43.8%
	Air conditioner disinfectant	Aerosol	23.8%	16.2%	82.1%	58.3%
	Chlorine-based disinfectant	Tablet	68.3%	28.1%	79.0%	67.7%
Repellent	Fabric repellent	Trigger	22.5%	17.4%	72.5%	62.7%
	Insect repellent for food	Tablet	13.4%	4.5%	30.0%	55.9%
Preservative	Wood preservative	Aerosol	75.0%	43.8%	93.8%	56.3%

**Table 5:** Summary of the reported product use and frequency of use.

Product	Product Use Rate (%)		Frequency of Use (Use/Month)		
	Current Study	California *	Current Study	California *	The Netherlands **
Drain disinfectant (Algae remover)	38.2%	-	2.38 ^t^, 2.90 ^ta^	-	3/year
Insect repellent	9.7%	46% ^♂^, 47% ^♀^	0.4	1.6 ^♂^, 2.4 ^♀^	54/year
Wood preservative	1.1%	-	0.1	-	-

* [14], ** [24]; ^t^ trigger, ^ta^ tablet; ^♂^ Men; ^♀^ Women.
